# Premature diagnostic closure after pericarditis treatment delays recognition of ATTR cardiomyopathy: a multimodality case report

**DOI:** 10.1093/ehjcr/ytag310

**Published:** 2026-05-05

**Authors:** Christopher J Kocx, Zara Rolfe, Jo-Dee Lattimore, Imre Hunyor

**Affiliations:** Department of Cardiology, Royal Prince Alfred Hospital, Camperdown, Sydney, NSW 2050, Australia; Faculty of Medicine and Health, The University of Sydney, Sydney, NSW 2000, Australia; Department of Cardiology, Royal Prince Alfred Hospital, Camperdown, Sydney, NSW 2050, Australia; Department of Cardiology, Royal Prince Alfred Hospital, Camperdown, Sydney, NSW 2050, Australia; Faculty of Medicine and Health, The University of Sydney, Sydney, NSW 2000, Australia; Department of Cardiology, Royal Prince Alfred Hospital, Camperdown, Sydney, NSW 2050, Australia; Faculty of Medicine and Health, The University of Sydney, Sydney, NSW 2000, Australia

**Keywords:** Cardiac amyloidosis, ATTR cardiomyopathy, Pericarditis, Troponin, Multimodality imaging, Diagnostic closure, Case report

## Abstract

**Background:**

Cardiac amyloidosis can present with troponin-positive chest pain and non-obstructive coronary arteries, mimicking acute coronary syndrome. When a concurrent treatable diagnosis, such as pericarditis, is identified, symptomatic improvement may lead to premature diagnostic closure and failure to pursue confirmatory testing for an underlying infiltrative cardiomyopathy.

**Case summary:**

A man in his 70s presented with pleuritic chest pain and troponin-T of 1840 ng/L. Coronary angiography was normal. Echocardiography revealed left ventricular hypertrophy with apical sparing on longitudinal strain. Cardiac magnetic resonance showed diffuse subendocardial late gadolinium enhancement and elevated extracellular volume (48%). Positron emission tomography (18F-FDG-PET) confirmed active pericarditis but no myocardial inflammation. Endomyocardial biopsy confirmed amyloid deposits; 99mTechnetium pyrophosphate scintigraphy confirmed transthyretin amyloid (ATTR) cardiomyopathy. He was treated for myopericarditis and referred for ATTR therapy.

**Discussion:**

This case highlights how symptomatic resolution after treatment of acute pericarditis can obscure a co-existing infiltrative cardiomyopathy, and why persistent imaging red flags, including apical sparing on strain, diffuse subendocardial late gadolinium enhancement, and markedly elevated extracellular volume, should prompt definitive testing regardless of clinical improvement.

Learning points

- When a concurrent acute diagnosis, such as pericarditis, responds to treatment, clinicians should pursue confirmatory testing for infiltrative cardiomyopathy if imaging red flags persist.
- Persistent biomarker elevation should prompt investigation for an alternative or co-existing substrate, such as infiltrative cardiomyopathy.

## Introduction

Troponin-positive chest pain with unobstructed coronary arteries (MINOCA) is common and diagnostically treacherous. The differential spans coronary plaque disruption without angiographic stenosis, spasm, microvascular dysfunction, myocarditis, Takotsubo syndrome, and infiltrative cardiomyopathy. Cardiac amyloidosis can present with chest pain, disproportionate biomarker elevation, and hypertrophy with preserved ejection fraction and may be overlooked when an alternative acute diagnosis is apparent.^1^ We report a case in which treatment of acute pericarditis resolved the presenting symptoms, but persistent imaging abnormalities prompted further investigation that ultimately revealed transthyretin amyloid (ATTR) cardiomyopathy as the underlying substrate. This case illustrates the risk of premature diagnostic closure and the importance of pursuing red flags even after an acute diagnosis has been treated.

## Summary figure

**Figure:**
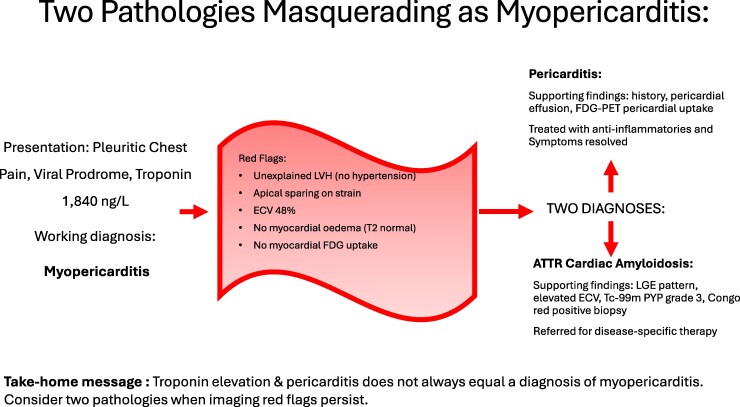


## Case presentation

A 70-year-old man presented with acute pleuritic chest pain after a 1-week viral prodrome. He also described 6 months of progressive exertional dyspnoea previously attributed to deconditioning. He was haemodynamically stable (blood pressure 150/90 mmHg). Examination showed elevated jugular venous pressure with otherwise normal heart sounds, no peripheral oedema, and no classical amyloid stigmata. He was initially managed via an acute coronary syndrome (ACS) pathway.

He had hypercholesterolaemia and two prior cryptogenic embolic strokes, with no hypertension, diabetes, smoking, or family history of cardiomyopathy; his medications included statin and antiplatelet therapy.

The primary differential was non-ST elevation myocardial infarction (NSTEMI) given the markedly elevated troponin. However, the pleuritic nature of the pain and viral prodrome raised suspicion for acute myopericarditis. The subacute dyspnoea and unexplained left ventricular hypertrophy (LVH) broadened the differential to include infiltrative cardiomyopathies. Pulmonary embolism and aortic dissection were excluded based on clinical scoring and D-dimer. The working diagnosis was troponin-positive chest pain with non-obstructive coronary arteries, initially managed along a MINOCA pathway, requiring differentiation between inflammatory and infiltrative aetiologies.

The electrocardiogram (ECG) (*Figure 1*) on presentation showed sinus rhythm with no ST-segment elevations or depressions. There were no dynamic ischaemic changes on serial ECGs. QRS amplitudes appeared disproportionately modest relative to the degree of ventricular hypertrophy observed on imaging, suggestive of voltage–mass discordance. Pseudo-infarct patterns (Q waves) were not present. High-sensitivity troponin-T was 1840 ng/L (normal < 14 ng/L), prompting ACS protocol activation. C-reactive protein was elevated (90 mg/L). Renal function was normal.

**Figure 1:**
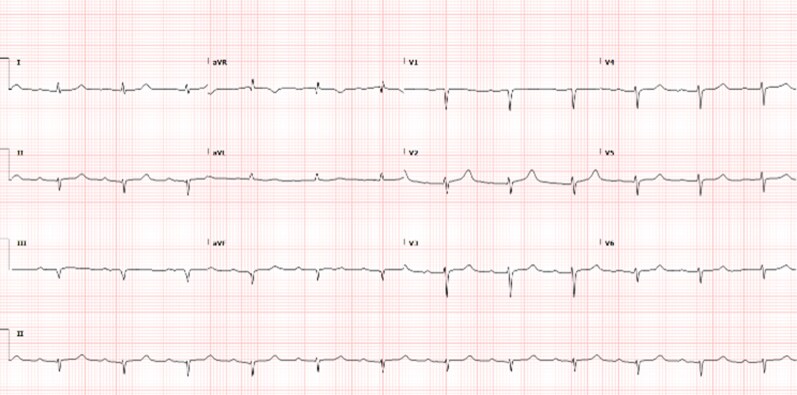
Electrocardiogram on presentation. Twelve-lead ECG showing sinus rhythm. QRS amplitudes are disproportionately modest relative to the significant left ventricular hypertrophy seen on imaging, suggestive of voltage–mass discordance. There are no acute ST-segment deviations suggestive of ischaemia.

Transthoracic echocardiography (*Figure 2*) showed preserved ejection fraction (60%) with concentric biventricular hypertrophy (septum 15 mm). Both atria were enlarged, and a small posterior pericardial effusion was noted. Doppler analysis revealed Grade II diastolic dysfunction. Retrospective speckle-tracking analysis demonstrated reduced global longitudinal strain (−9.1%) with relative apical sparing.

**Figure 2:**
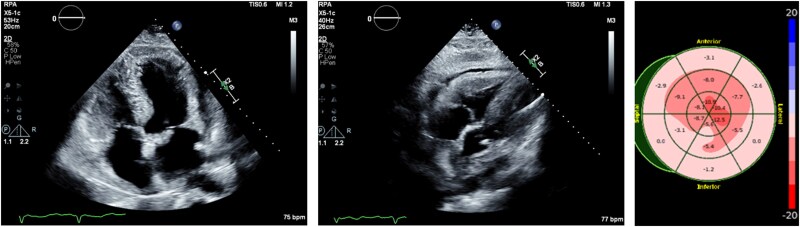
Transthoracic echocardiography: (*A*) apical four-chamber: demonstrating left ventricular hypertrophy, biatrial enlargement, thickening of the interatrial septum, and trace pericardial effusion. (*B*) Subcostal four-chamber: demonstrating marked right ventricular hypertrophy and trace pericardial effusion. (*C*) Global longitudinal strain plot showing reduced global longitudinal strain with relative preservation of apical segments compared to basal segments.

Given the positive troponin, the patient underwent urgent invasive coronary angiography (*Figure 3*). This revealed non-obstructed coronary arteries with only minimal atherosclerosis. No culprit lesion or thrombosis was identified.

**Figure 3:**
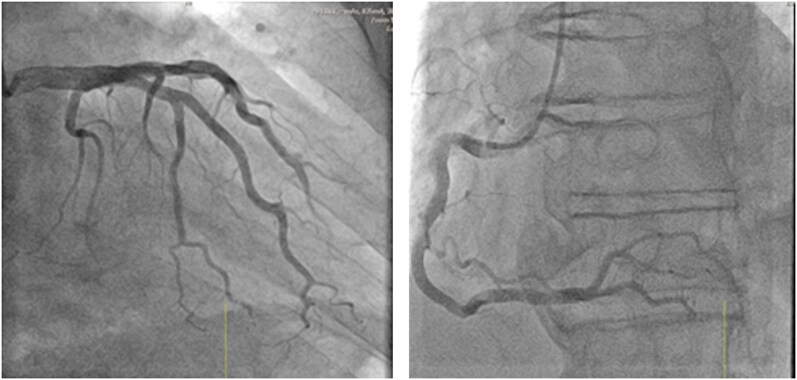
Coronary angiography. (*A*) RAO caudal view showing no obstructive disease or thrombus. (*B*) LAO view showing no obstructive disease or thrombus.

A 3 T cardiac magnetic resonance image (MRI) was performed for tissue characterization (*Figure 4*). This showed marked thickening of the interventricular septum and right ventricle (RV) free wall, with preserved systolic function. There was diffuse subendocardial late gadolinium enhancement (LGE) extending into patchy transmural areas. T1 mapping revealed a diffusely elevated native T1 time, and the calculated extracellular volume (ECV) was 48%. Native T2 mapping was normal, indicating no significant myocardial oedema. An incidental 2.5 cm right ventricular mass was also noted, with signal characteristics on cardiac magnetic resonance (CMR) consistent with benign cardiac fibroma.

**Figure 4:**
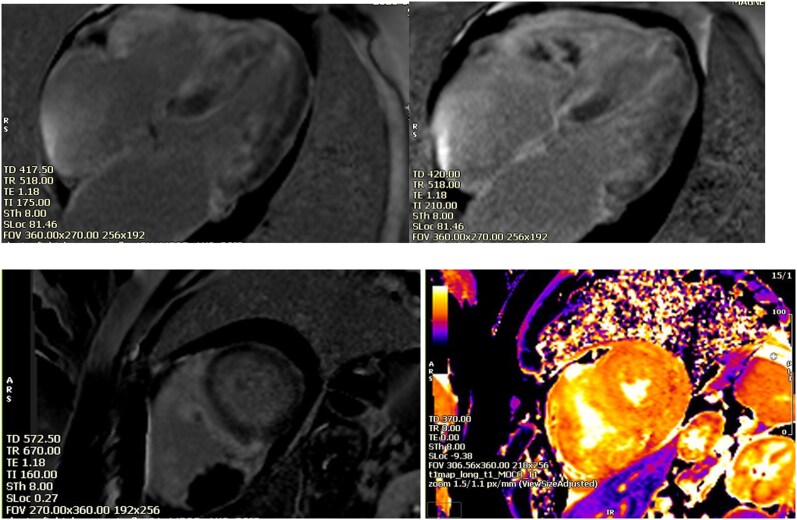
Cardiac magnetic resonance imaging. (*A*) Four-chamber late gadolinium enhancement imaging demonstrating diffuse subendocardial enhancement. (*B*) Four-chamber late gadolinium enhancement view showing diffuse subendocardial enhancement and an incidental right ventricular mass that is hypointense on late gadolinium enhancement, consistent with a cardiac fibroma. (*C*) Short-axis late gadolinium enhancement image confirming diffuse circumferential subendocardial enhancement with patchy transmural extension. (*D*) Native T1 map demonstrating diffusely elevated myocardial T1 values; the calculated extracellular volume was 48% (normal 25%–30%).

Given the diagnostic uncertainty between myocarditis and infiltrative disease, particularly the discordance between elevated troponin and normal T2 mapping, an positron emission tomography (18F-FDG-PET) with myocardial uptake suppression protocol was performed. FDG-PET is not part of routine workup in this context but was pursued to differentiate myocarditis from isolated pericarditis and to characterize the incidental RV mass. The PET showed focal uptake in the pericardium and moderate uptake in the right atrial wall, consistent with active pericarditis. Importantly, there was no diffuse myocardial FDG uptake, arguing against active myocarditis in the ventricular myocardium. The PET findings supported a diagnosis of pericarditis superimposed on an infiltrative cardiomyopathy. However, as PET could not identify the nature of the infiltrative substrate, endomyocardial biopsy was performed from the RV septum (*Figure 5*). Histopathology demonstrated Congo red positive deposits with apple-green birefringence under polarized light, confirming amyloid protein deposition in the myocardium (*Figure 5A*). Electron microscopy revealed characteristic non-branching fibrils ∼8–10 nm in diameter in a random extracellular distribution, consistent with amyloid (*Figure 5B*). This provided a definitive tissue diagnosis of cardiac amyloidosis.

**Figure 5:**
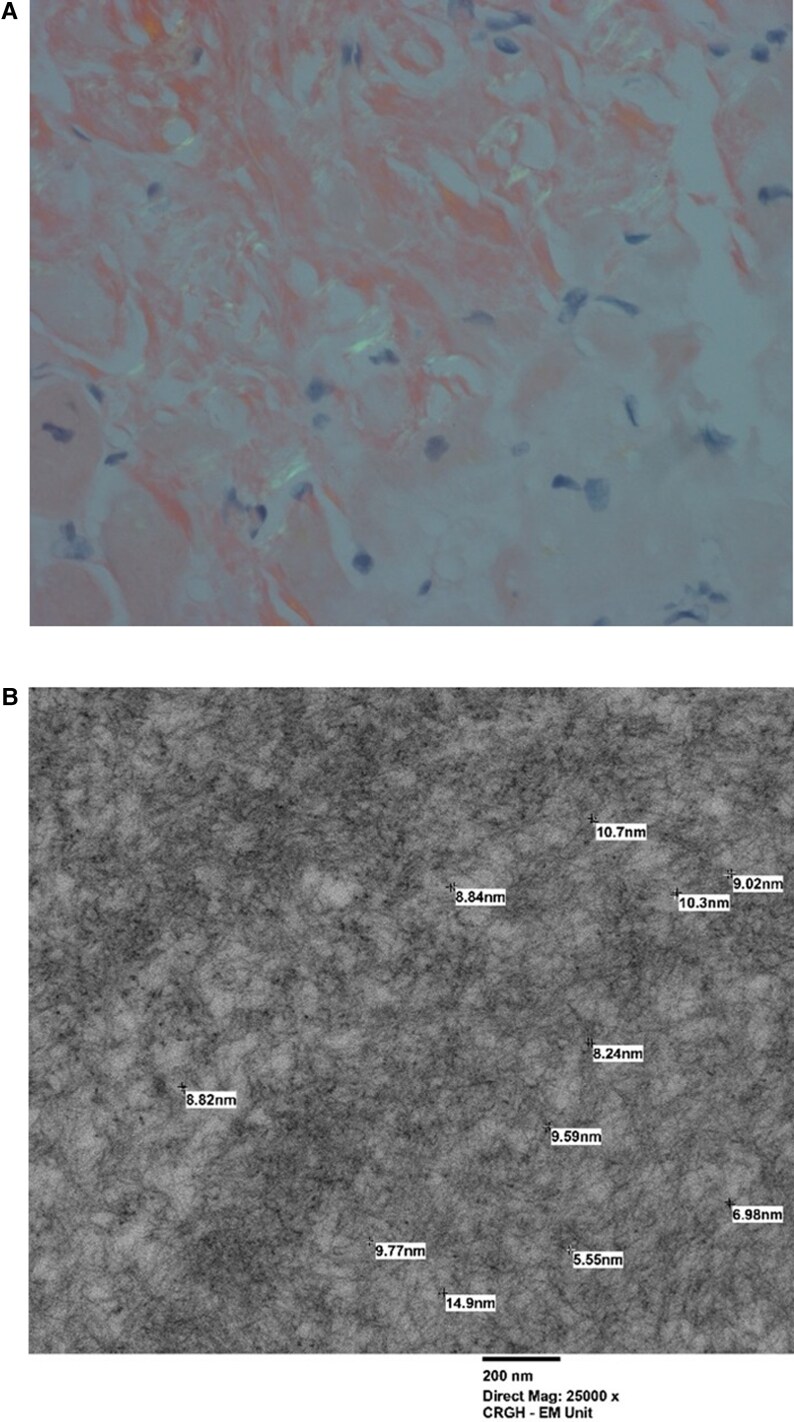
Endomyocardial biopsy: (*A*) right ventricular biopsy with Congo red staining under polarized light. (*B*) High-magnification electron micrograph showing randomly arranged, non-branching fibrils ∼8–10 nm in diameter.

### Management

The patient was initially treated for myopericarditis with ibuprofen (600 mg thrice daily (TDS)) and colchicine (0.5 mg twice daily (BD), later reduced and then stopped due to gastrointestinal intolerance). Prednisolone was started for ongoing symptoms (25 mg daily, weaning) with chest pain resolving over 3 days. Telemetry detected atrial fibrillation, and anticoagulation was commenced.

### Outcome and follow-up

The patient’s hospital course was uncomplicated after therapy was started. He was discharged home pain-free. Serum and urine immunofixation and free light-chain assays were negative for light-chain (AL) amyloidosis. This finding, combined with the patient’s demographic and cardiac features, raised suspicion for ATTR amyloidosis.

As an outpatient, the patient underwent a 99mTechnetium pyrophosphate (Tc-99m PYP) scintigraphy scan. The scan demonstrated intense radiotracer uptake in the myocardium (Grade 3 Perugini uptake), confirming a diagnosis of transthyretin cardiac amyloidosis. The patient was commenced on tafamidis (61 mg daily). Genetic testing to distinguish hereditary from wild-type ATTR was performed, and results are pending at the time of writing.

At 1-month follow-up, troponin-T had declined from 1840 ng/L at presentation to 95 ng/L, still elevated above the upper limit of normal (<14 ng/L). At 10-month follow-up, the patient remained in New York Heart Association (NYHA) functional Class II with stable exertional dyspnoea on tafamidis therapy. Troponin-T remained mildly elevated at 45 ng/L.

## Discussion

### Diagnostic reasoning and the risk of premature closure

The principal teaching point of this case is not the imaging red flags of cardiac amyloidosis *per se*; apical sparing on strain, diffuse subendocardial LGE, elevated ECV, and voltage–mass discordance are well-established diagnostic features.^2–4^ Rather, this case illustrates how identification and successful treatment of an acute concurrent diagnosis (pericarditis) can lead to premature diagnostic closure, delaying recognition of an underlying infiltrative cardiomyopathy. The patient’s chest pain resolved with anti-inflammatory therapy and troponin declined, creating a plausible narrative of uncomplicated myopericarditis. It was the persistence of imaging abnormalities, wall thickening disproportionate to haemodynamic load, severely reduced global longitudinal strain (GLS) with apical sparing, markedly elevated ECV, and the absence of myocardial oedema on T2 mapping that compelled further investigation and ultimately led to tissue diagnosis. Amyloid infiltration likely provided the substrate for chronic myocardial injury, with viral pericarditis superimposing an acute inflammatory component that both elevated troponin and produced pleuritic symptoms mimicking infarction.^1,5^

A definitional note is warranted. Although this patient was initially managed along a MINOCA pathway, the subsequent identification of pericarditis with myocardial injury raises the question of whether this presentation meets the strict European Society of Cardiology (ESC) definition of MINOCA, which implies an ischaemic mechanism.^6^ The final diagnosis is more accurately described as troponin-positive myocardial injury with a dual substrate: chronic amyloid infiltration and acute pericardial inflammation. We retain reference to the MINOCA pathway because it reflects the real-world clinical approach with coronary angiography followed by systematic tissue characterization and because infiltrative cardiomyopathy should remain in the differential for any troponin-positive presentation with non-obstructive coronary arteries.

The troponin-T of 1840 ng/L merits specific comment. In ATTR cardiomyopathy, chronic low-grade troponin elevation is common and correlates with disease severity and prognosis, typically in the range of 50–200 ng/L.^1^ Acute pericarditis or myocarditis can produce transient elevations into the thousands. The magnitude of elevation in this case likely reflects an acute inflammatory component superimposed on a background of chronic amyloid-related myocardial injury. No prior troponin values were available for comparison. However, the subsequent trajectory, declining from 1840 to 95 ng/L at 1 month with anti-inflammatory therapy, and then plateauing at 45 ng/L at 10 months despite clinical stability on tafamidis, is consistent with this dual-substrate model. The initial decline reflects resolution of the acute inflammatory component, while the persistent elevation reflects ongoing amyloid-related myocardial injury.^1,4^

### Overlap of amyloidosis and myopericarditis

Pericardial effusions are not uncommon in cardiac amyloidosis, but clinically overt pericarditis confirmed by FDG-PET as in this case is distinctly uncommon and, to our knowledge, has been rarely reported in transthyretin amyloid related cardiomyopathy (ATTR-CM).^7^ The causal relationship is uncertain as pericardial amyloid infiltration may lower the threshold for inflammatory pericarditis in the setting of a viral trigger, though this remains speculative. The clinical consequences are clear, as symptomatic improvement with anti-inflammatory therapy risks anchoring to a purely inflammatory diagnosis. In amyloidosis, troponin and natriuretic peptide (NT-proBNP) may remain chronically elevated, unlike the transient biomarker profile expected in uncomplicated pericarditis or other acute aetiologies.^1,4^ Without tissue confirmation and follow-up surveillance, the underlying diagnosis, and access to disease-modifying therapy with transthyretin stabilizers, could have been missed, with significant prognostic implications given the median survival of untreated ATTR-CM.

## Conclusion

This case demonstrates that successful treatment of an acute concurrent diagnosis should not preclude investigation of persistent imaging abnormalities. In troponin-positive presentations with non-obstructive coronary arteries, imaging hallmarks of infiltrative disease should prompt confirmatory testing with pyrophosphate scintigraphy or endomyocardial biopsy, even when symptoms have resolved and an alternative acute diagnosis has been established.

## Data Availability

The data underlying this case report are not publicly available due to patient privacy considerations. De-identified data may be made available from the corresponding author upon reasonable request.

## Lead author biography

Dr Christopher J. Kocx is a cardiologist and cardiac imaging fellow in Sydney, Australia, and a PhD candidate at the University of Sydney. His research focuses on right heart adaptation and maladaptation, integrating echocardiography, cardiac MRI, haemodynamics, and translational tissue-based analyses to improve risk stratification and mechanistic understanding in pulmonary hypertension, tricuspid regurgitation, and heart failure. He has a strong interest in multimodality imaging in complex diagnostic presentations and in developing practical imaging pathways that translate into better clinical decision-making and patient outcomes.

## Notes

**Consent:** Written informed consent was obtained from the patient for the publication of this case report and the accompanying images.
